# Direct Environmental Lead Detection by Photoluminescent Perovskite Formation with Nanogram Sensitivity

**DOI:** 10.1021/acs.est.3c06058

**Published:** 2023-11-27

**Authors:** Lukas Helmbrecht, Sjoerd W. van Dongen, Arno van der Weijden, Christiaan T. van Campenhout, Willem L. Noorduin

**Affiliations:** † AMOLF, Science Park 104, Amsterdam 1098 XG, The Netherlands; ‡ Van ‘t Hoff Institute for Molecular Sciences, University of Amsterdam, Science Park 904, Amsterdam 1090 GD, The Netherlands; ¶ Lumetallix B.V, Science Park 104, 1098 XG Amsterdam, The Netherlands

**Keywords:** lead detection, lead pollution, perovskite, photoluminescence, lead paint, lead glazing

## Abstract

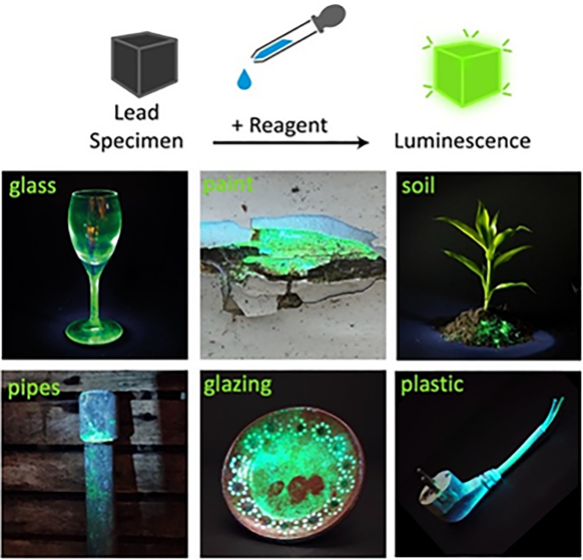

Although the global ban on leaded gasoline has markedly reduced lead poisoning, many other environmental sources of lead exposure, such as paint, pipes, mines, and recycling sites remain. Existing methods to identify these sources are either costly or unreliable. We report here a new, sensitive, and inexpensive lead detection method that relies on the formation of a perovskite semiconductor. The method only requires spraying the material of interest with methylammonium bromide and observing whether photoluminesence occurs under UV light to indicate the presence of lead. The method detects as little as 1.0 ng/mm^2^ of lead by the naked eye and 50 pg/mm^2^ using a digital photo camera. We exposed more than 50 different materials to our reagent and found no false negatives or false positives. The method readily detects lead in soil, paint, glazing, cables, glass, plastics, and dust and could be widely used for testing the environment and preventing lead poisoning.

## Abstract 2

There is an urgent need for the detection of lead pollution. This study introduces a new lead detection method for the direct rapid testing and mapping of the environment.

## Introduction

The chemical element lead is toxic: short-term exposure to a low dosage of lead can inflict permanent damage with immediate danger for life and health.^1−3^ Lead poisoning is most harmful for young children and can cause lifelong severe health problems, ranging from decreasing neurological and cognitive functions such as loss of IQ, behavioral problems, aggression, and learning disabilities, to severe physical illnesses such as blindness, convulsions, and death.^1−3^ Due to the omnipresence of lead in our environment—ranging from water pipes, cables and paints, to glassware, jewelry, electronics, mining and recycling, UNICEF estimates that more than one out of three children worldwide (800 million children) suffer from lead poisoning, and the cost of lead poisoning is estimated at 6.9% of the Gross National Product every year.^1−16^ Moreover, unlike organic pollutants, lead is not degradable and can therefore remain present to inflict harm for decades or longer.^1−5^

To prevent lead poisoning, locating lead is the essential first step.^1−3^ Already, many lead detection methods have been developed, ranging from precise analytical methods such as atomic absorption spectroscopy (AAS) and X-ray fluorescence spectroscopy (XRF), to DNA fluorescence reactions and simple color tests for field testing and community science.^17−22^ Despite this progress, none of these methods offer quick, large-area detection of lead with high sensitivity, which is highly desirable for many practical lead detection scenarios, as it is often unclear where lead is located. Reflecting these shortcomings, the environment is often tested for lead only after elevated levels of lead have been found in the blood of children, when it is too late and exposure has already occurred.^2^ These insights highlight the urgent need for a sensitive, accurate, and low-cost lead detection method. Moreover, lead pollution is oftentimes very heterogeneous.^23^ Therefore, it would be highly beneficial to directly visualize lead, with numerous tests covering large areas.

We here develop a lead detection method that is based on the formation of light-emitting lead halide perovskite semiconductors. Lead halide perovskites have received tremendous attention in recent years due to their extraordinary chemical, optical, and electronic performance, and many synthesis methods have been developed for applications such as catalysis, solar cells, and LEDs.^24−32^ Recently, the formation of photoluminescent lead halide perovskites has also been used as a method to indicate the presence of lead in water and oils,^29,33^ yet the requirement to take samples for isolating, concentrating, and purifying the lead highlights that a fundamentally different approach is needed for direct detection of lead in the environment.

**Figure 1:**
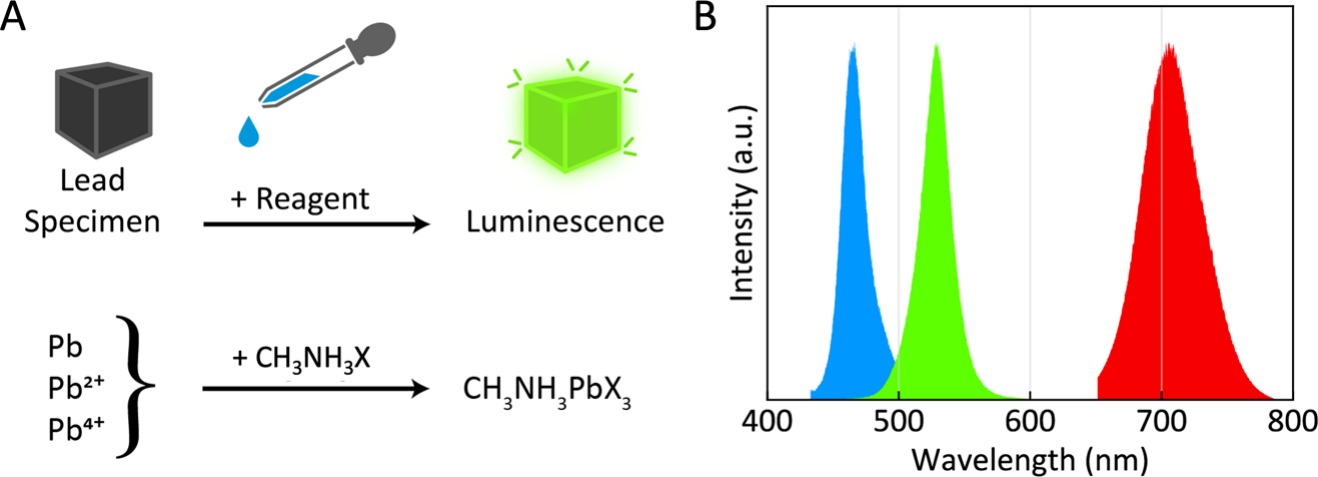
Concept of photoluminescent lead detection. (A) A reagent containing a perovskite precursor (here methylammonium halide (CH_3_NH_3_X, X = Cl, Br, I); see Supporting Information for other precursors) reacts with different forms of lead (Pb, Pb^2+^, Pb^4+^) to yield a perovskite (CH_3_NH_3_PbX_3_) that shows photoluminescence (PL) to indicate the presence of lead. (B) Selection of the halide moiety enables tuning of the PL signal by mixing halides in the reagent in different ratios: Cl:Br (6:4) for blue, Br for green, or Br:I (2:8) for red PL. Colors of the graphs indicate the emission colors of the maximum intensity of the PL signal. Since the human eye is most sensitive to green light, methylammonium bromide (CH_3_NH_3_Br) is selected as reagent for most practical lead detection situations.

The objective of this study is to develop a direct environmental lead testing method based on perovskites, benchmark the chemoselectivity and sensitivity, and explore the application potential in real-world scenarios. Our test is performed on a specimen by direct application of a reagent that converts lead into a perovskite semiconductor (Figure 1). Under an ultraviolet (UV) flashlight, the perovskite emits photoluminesence (PL) strongly in the visible range at a wavelength that can be tuned by adjusting the halide moiety to facilitate detection with the naked eye (Figure 1). The reagent is nontoxic, stable, and inexpensive. Remarkably, the reagent directly converts lead into a light-emitting perovskite in a wide selection of specimens ranging from plastics and paints, to glazing and glass. Importantly, lead specimens with different oxidation states, and with different counterions, all react to form the same photoluminescent perovskite, while other metals such as tin do not react, such that the lead test offers strong versatility and selectivity.

## Materials and Methods

### Reagent Selection

Based on our experience with conversion reactions,^34−39^ we screened an extensive combination of perovskite precursors, solvents, and application methods. The reagents include organic methylammonium, phenethylammonium, butylammonium, and formamidinium halides as well as inorganic cesium halides in a variety of solvents. Additionally, the PL color is tunable by adjustment of the halide moiety (see Figure 1B). We selected methylammonium bromide (CH_3_NH_3_Br) dissolved in isopropanol (IPA) as a reagent that is readily available, relatively safe (corrosive, not toxic), and cheap, and because the human eye is most sensitive to green light emission from the resulting methylammonium lead bromide perovskite (CH_3_NH_3_PbBr_3_). Isopropanol of 99.9% purity is used to avoid the excess presence of water, which can disturb the reaction. Note that methylammonium bromide is corrosive, isopropyl alcohol is flammable, and UV light is harmful to one’s eyes. Therefore, for practical application of this testing method we recommend the following precautions: (1) wear safety glasses; (2) use in well-ventilated spaces; (3) keep reagent away from sparks, open flames, and other ignition sources, (4) test on less visible areas and wash afterward with water, and (5) do not look in the UV light. We find that the reagent can be applied by simply dripping, spraying, rubbing, or brushing the reagent on, but for most applications, and area mapping using a spray dispenser to coat the specimen is most practical.

### Sensitivity Testing

We benchmark the sensitivity of our method by measuring the PL intensity for different concentrations of lead. To this aim, we loaded different concentrations of lead in a matrix material. Based on preliminary screening, we find that diatomaceous earth is a suitable matrix material because it is optically transparent and nonfluorescent while also offering a high surface-to-volume ratio for absorbing the lead. To determine the sensitivity, different amounts of lead acetate trihydrate (Pb(II)(OAc)_2_)·3H_2_O (91 pg/mm^2^ to 18 μg/mm^2^, for a total of 50 pg/mm^2^ to 10 μg/mm^2^ lead) are applied to circles (ø3.2 mm) of diatomaceous earth and irradiated with UV light (365 nm LED) after drying. Subsequently, we drip CH_3_NH_3_Br in IPA (5 μL, 0.16M) on the circle and record the PL signal using a Canon EOS 800D photo camera (see Supporting Information for details). To compare our test against the state-of-the-art in coloring reactions, we used two commonly used commercially available rhodizonate tests (LeadCheck instant lead test made by 3M, and a widely sold generic sodium rhodizonate test) and recorded the color change upon contact with the lead-acetate infused circles. Moreover, we analyzed the circles through XRF (see Supporting Information).

### Chemoselectivity

To determine the chemical selectivity of the method, the PL was recorded after the CH_3_NH_3_Br reagent was applied to more than 50 different materials that were either lead-free or contained lead (see Supporting Information for a full list). In particular we select metals such as tin and tin salts, as these have historically been confused with lead.

### Detection in Complex Media and Real-World Scenarios

To explore the potential of lead testing in complex and real-world scenarios, we apply the reagent using a spray bottle on lead-containing specimens, such as paints, glassware, pottery, soil, batteries, and electrical cables, and record the emitted PL using photography and videos. To verify the lead content in these specimens, we perform additional analysis using XRF (see Supporting Information for details).

## Results and Discussion

### Lead Testing with Nanogram Sensitivity

**Figure 2:**
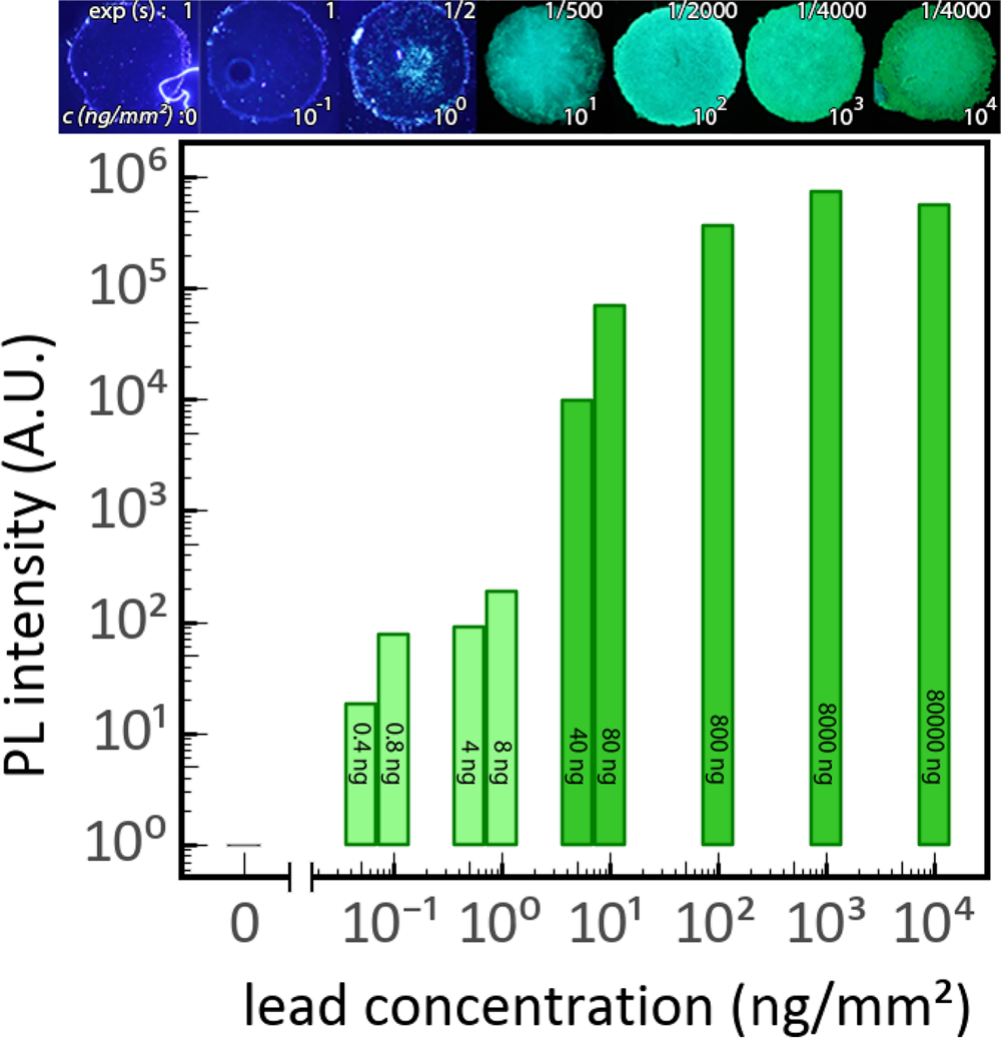
Sensitivity of photoluminescent lead detection: Photographs and graph of maximum PL intensity after reacting different concentrations of lead acetate on ø3.2 mm circles of diatomaceous earth with 5 μL CH_3_NH_3_Br reagent, showing visible lead detection down to ca. 1 ng/mm^2^ by the naked eye and 50 pg/mm^2^ using image processing. The total weight of applied lead is denoted inside the bars. Note that the shutter time has been adjusted for each photograph to prevent overexposure, such that their respective brightness does not directly relate to the measured PL.

The testing method shows a sigmoidal-like response in PL as a function of the concentration of lead. The brightness is lower at lower concentrations of lead, and the PL dims over time, but the PL, and therefore the presence of lead, is easily detectable even by the naked eye (Figure 2). Bright PL over the entire sample is immediately visible down to concentrations of 10 ng/mm^2^, while PL becomes inhomogeneous but is still clearly visible down to lead concentrations of 1 ng/mm^2^. More so, by optimizing the shutter time of a photo camera and performing postprocessing, it is possible to detect 50 pg/mm^2^ of lead under this specific scenario. An additional set of experiments was performed under constant camera settings, which confirmed the reproducibility of the sigmoidal-like behavior and detection limit of nanograms per square millimeter (see Supporting Information for details).

The commercial coloring reactions based on rhodizonate show a color change from yellow to red when testing positive for lead. We find that for lead concentrations of 1 μg/mm^2^ and higher we can clearly observe the formation of the red-colored lead-rhodizonate complex, but below these concentrations the color is hard to interpret (see Supporting Information). Hence, our luminescent lead testing method has a more than 1000 times increase of sensitivity compared to the current state-of-the-art in coloring tests. This increased sensitivity is at least partially explained by the fundamentally different detection mechanism of our photoluminescent method: the coloring reactions are based on absorption of light and turn lead into muted colors such as brown, off-yellow, or red, which makes interpretation of results difficult. In contrast, our luminescent lead testing method is based on the emission of light in bright and highly visible colors, which makes detection of nanograms of lead straightforward. XRF measurements confirm the applied lead concentrations (see Supporting Information). Moreover, we observe that for these specific kind of samples our test is more sensitive than the XRF measurements.

### High Chemoselectivity

**Figure 3:**
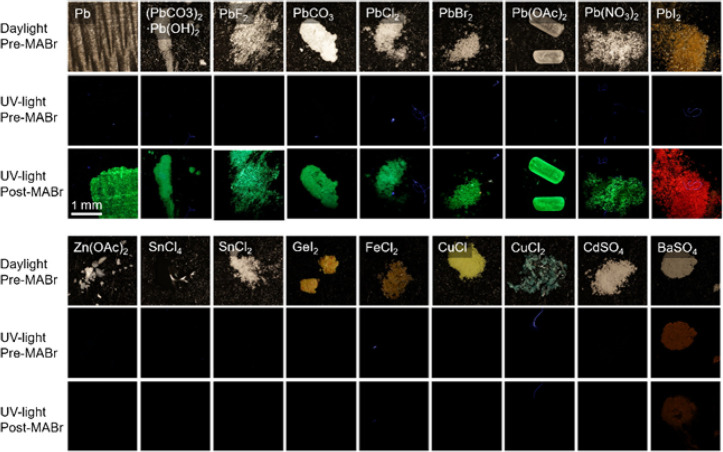
Chemoselectivity of photoluminescent lead detection: Selection of lead-containing, and lead-free samples before conversion under daylight, UV-light, and after spraying with the CH_3_NH_3_Br reagent, showing that all lead-containing samples show PL after conversion, while none of the lead-free samples show PL (an extensive list of more than 50 compounds is given in the Supporting Information). Lead-containing samples with halides can cause color shifts in the PL signal (PbF_2_ blueish, PbI_2_ red).

For determining the chemoselectivity, we clearly observe a PL signal for all tested lead containing specimens, although PL intensity varies between different compounds and can change over time (Figure 3, see Supporting Information for an extended list). The presence of halides in the specimen can shift the PL wavelength: lead iodide gives red PL, which is consistent with the formation of the corresponding mixed perovskites (Figure 1). Given that the reaction toward the perovskite requires Pb^2+^, it is surprising that other oxidation states of lead still react toward photoluminescent perovskites. We hypothesize that these different oxidation states of lead first undergo a redox reaction with the reagent or environment to form Pb^2+^, which subsequently reacts into the desired perovskite. For instance, metallic lead (Pb^0^) may readily oxidize in the air into Pb^2+^, which subsequently can react with CH_3_NH_3_Br to yield the perovskite, while Pb^4+^ undergoes reductive dissolution in traces of water to yield Pb^2+^.^39,40^ Hence, all oxidation states of lead can be detected via reaction into photoluminescent perovskites.

In contrast, none of the lead-free specimens produced any PL after contact with the reagent. As expected, different forms of commonly used metals such as copper, zinc, and iron do not show PL (Figure 3). Barium sulfate shows slight fluorescence under UV irradiation before the addition of CH_3_NH_3_Br, but no noticeable change is observed after application of the reagent. To exclude false positives, we investigated different forms of tin. Tin has very similar physical and chemical properties to lead, and photoluminescent tin perovskites have previously been reported.^37^ Despite these similarities with lead, we find that none of the tin specimens that we tested produce any PL signal after contact with the reagent. Compared to rhodizonate coloring reactions, our photoluminescent lead testing method shows better chemoselectivity, as rhodizonate tests are known to give false positives with both barium and antimony.^41,42^ Overall, these results show that our reagent exclusively results in photoluminescence with a lead-containing specimen, indicating that both false negative and false positive detection of lead is highly unlikely.

### Direct Lead Detection in Complex Media and Real-World Scenarios

**Figure 4:**
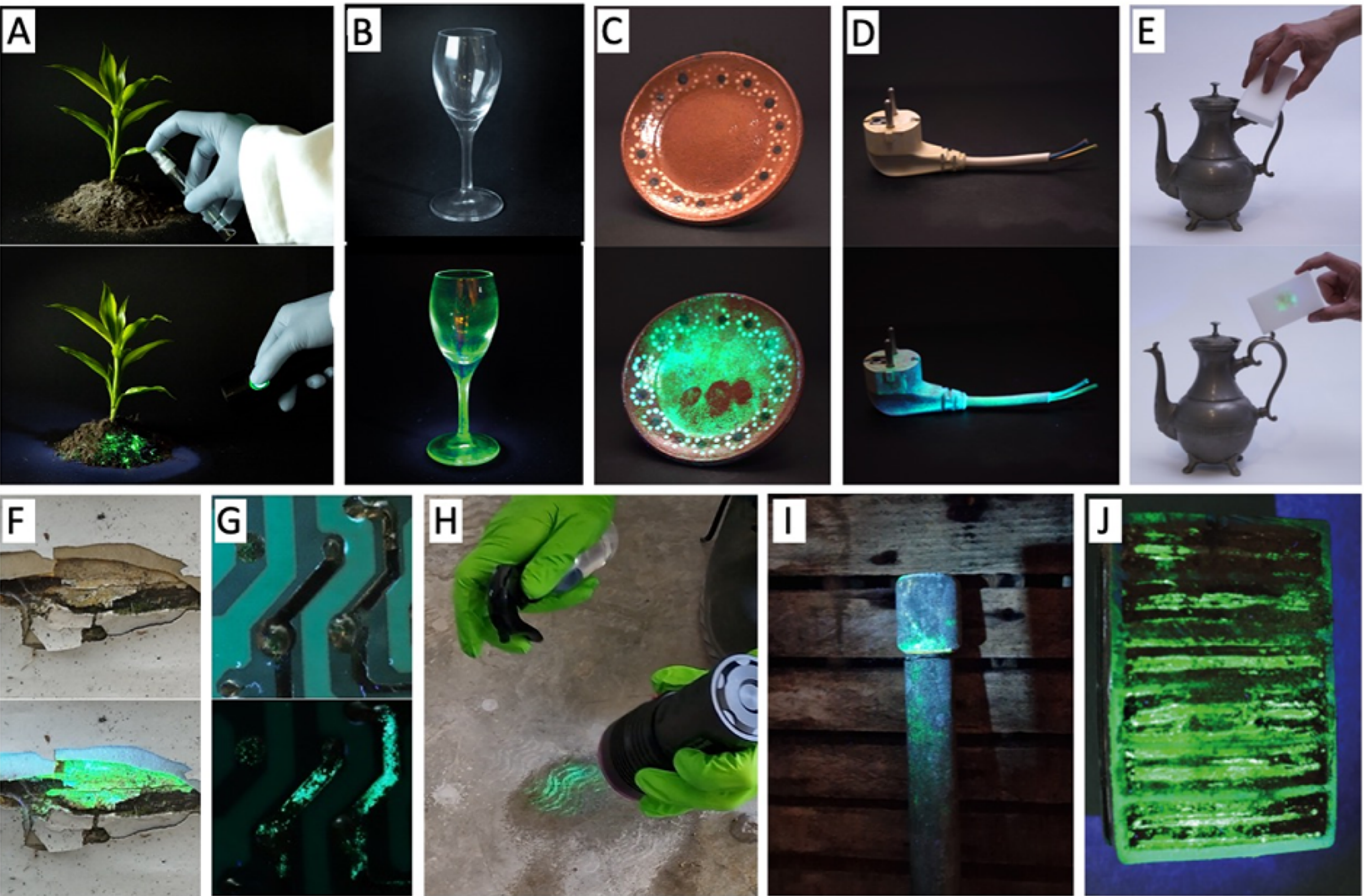
Photoluminescent lead detection in complex environments and real-world scenarios. Lead detection on (A) soil, (B) glass, (C) pottery, (D) electrical cable cladding, (E) lead-containing tea kettle that is rubbed with melamine sponge, (F) paint, (G) electrical circuit board, (H) footprint of lead-contaminated shoe, (I) waterpipe, and (J) cross-sectioned lead-acid battery. Note that the photoluminescence is brighter in reality than in the photographs.

We find that spraying the reagent on specimen such as paints, glassware, pottery, soil, batteries, and electrical cables results in bright PL (Figure 4), which is consistent with the verified lead content using XRF (glass 191,835 ppm; plate 226,579 ppm; cable 1,989 ppm; circuit board 52,466 ppm; battery 400,189 ppm). Although lead in paints and cladding of electrical cables is encapsulated in a polymer matrix, we observe a bright PL immediately after application of the reagent. Similarly, lead in glass is directly made visible (Figure 4B). Since diffusion of the reagent is slow inside glass, we find that the PL signal can be enhanced by slightly scratching the glass to increase the surface area for reaction with the CH_3_NH_3_Br. With paint (Figure 4F), we can even identify which layers of paint contain lead and which do not.

In some cases, the brightness of the PL can be enhanced by pretreatment of the sample. For instance, an increase in PL can be acquired by employing strategies that maximize the surface area. Consistent with this idea, applying the reagent on a tea kettle containing lead results in a dull PL signal, but rubbing the metal with a melamine sponge and subsequently detecting the lead on the sponge yields a bright PL signal because the particles are smaller and well separated (Figure 4E). Additionally, pretreatment of specimens with acids and redox couples can optimize the reaction. For instance, for detection of lead in solder, applying the reagent directly reveals visible PL. However, tin reduces Pb^2+^ such that less Pb^2+^ is available for perovskite formation. This reduction reaction can be counteracted by pretreating the solder with peroxide or acid to oxidize both tin and lead and thereby enhance the PL signal (Figure 4G).

Lead detection is also possible in complex media that can hinder or degrade perovskite formation. For example, we find that the lead electrodes in a cross-sectioned lead/acid battery immediately light up despite the presence of strong acid in the battery (Figure 4I). In addition, although water is known to degrade perovskites, the reaction in lead-contaminated garden soil results in clearly visible PL of the lead pollution (Figure 4A).

The scalability of our testing method also enables easy and quick mapping of large areas for the presence of lead. For instance, by using an applicator with a wide spray, we can directly visualize lead pollution on floors that have been walked on with shoes that are contaminated with lead dust, resulting in photoluminescent footprints after reaction (Figure 4H). Besides mapping, these results also highlight the potential of our lead detection method for forensic and safety applications.

In summary, we here report a new lead detection method that enables rapid direct testing and mapping of a wide range of relevant scenarios ranging from paints, cables, waterpipes, dust, glass, and plastic. Unexpectedly, despite the notoriously problematic stability of perovskites in real-world environments, we discover that testing of real-world environments directly by forming perovskites is reliably possible. Moreover, surprisingly, different forms of lead, with different counterions and in different specimens, are all converted by a single reagent to a photoluminescent perovskite product.

There are still many fundamental questions regarding why this reaction works so well and for so many different species of lead. Our current hypothesis is that a complex cascade of ion-exchange, acid/base, and redox chemistry can take place in tandem with dissolution/recrystallization processes to funnel different species of lead into a single perovskite.^34,39^ For instance, lead may be freed up from the specimen under acidic conditions. Subsequently this lead can undergo a redox reaction to form Pb^2+^, which then can crystallize as lead halide. In contact with an excess of methylammonium halide, these crystals can almost instantaneously react to form perovskites. The excess of reagent and the evolution of methylamine may drive these reactions toward the formation of a perovskite. However, this is just one of the possible reaction routes, and other reactions are possible. Also, the role of the other chemical species remains to be further understood. Hence, the reaction is deceivingly simple to perform yet fascinatingly complex to understand.

### Environmental Implications

We here achieve direct environmental detection of lead by exploiting properties of lead and perovskites that traditionally are considered disadvantageous. Currently, widespread application of perovskites in photovoltaic applications is hampered because of a great need to replace lead with less harmful metals, but perovskites with bright photoluminescence can still only straightforwardly be made using lead.^27−29^ Moreover, lead is harmful because lead can both accept and donate electrons, resulting in versatile reactivity that can disrupt many biological processes. Paradoxically, we here leverage these seemingly unfavorable properties to form perovskites with bright PL from many different forms of lead using a single, simple reagent and thereby achieve direct environmental lead detection with high chemoselectivity and high sensitivity.

For specific scenarios, such as bright environments and low lead concentrations, it can be desirable to perform measurements under controlled illumination conditions. To offer a practical solution for these scenarios, we foresee that miniaturization of our current setup for quantitative measurements is desirable. Nevertheless, although quantification of lead concentrations is, to a degree, possible with this method, we believe that the sigmoidal-like response in PL makes the test currently most suitable for binary testing around a threshold value for lead. Preliminary results already show that we can tune the sensitivity of the detection threshold by modifying the composition of the reagent.

We note that the formation of photoluminescent lead halide perovskites has previously been used to detect lead in water, and oils,^29,33^ but unlike our strategy, these methods all require complicated sample pre- or postprocessing. Also, our photoluminescent lead testing method outperforms commercially available rhodizonate coloring tests with more than 1000 times higher sensitivity, superior chemoselectivity, and straightforward interpretation of results. Compared to analytical methods such as AAS and XRF our method offers complementary and synergistic advantages. With suitable precautions, we believe that the testing procedure can be performed safely as a part of community science. Moreover, we foresee that the cost per test may be comparable to those of current coloring reactions. Therefore, the simplicity, scalability, and speed of our luminescent lead testing method can be exploited to rapidly screen large areas for lead via mapping or test many samples with high sensitivity. For precise quantification of lead concentrations, our method may be used as an initial screening, which can readily be complemented with well-established quantification methods such as AAS and XRF.

Under well-controlled laboratory conditions, we can detect nanograms of lead per square millimeter with the naked eye. However, in complex real-world environments, this sensitivity will be different and may require further optimization. We envisage that the reagent can be further modified to offer wide tunability in sensitivity and selectivity for different lead-detection scenarios, by for instance selecting different perovskite precursors and additives for tailoring the oxidation state of lead, increasing the release of lead from specimens, accelerating the formation of perovskite, or mitigating perovskite degradation.

Further tuning of the testing method can be done for specific scenarios. For example, background fluorescence may hinder the interpretation of the signal, as is, for instance, the case for testing of turmeric spices, which show green fluorescence due to curcumin. We foresee that such complications can be overcome by, e.g., extracting the lead from the background or modifying the reagent composition to tune the PL emission wavelength away from the background fluorescence. Moreover, typically concentrations of lead are expressed as weight per volume. In contrast, our testing method analyzes exposed lead on surfaces. For materials such as paint, the perovskite formation and resulting PL will likely be dependent on factors such as the structure and matrix around the lead. Nevertheless, we foresee that it will be feasible to translate between the PL signal and concentrations per volume for a well-defined specimen.

Because of its selectivity, robustness, and ease-of-use, our direct photoluminescent lead detection method can be valuable for applications such as forensics. Moreover, we foresee opportunities for scalable high-throughput detection of lead pollution, where the simplicity and visibility of our method may directly impact the effectiveness of lead poisoning prevention campaigns by raising awareness of the presence of invisible lead pollution and guiding remediation operations. Steps in these directions are currently being taken.

## Supporting Information Available

Photoluminescent measurement setup, a list of tested materials and testing methods, solvent screening, different types of halides and halide mixtures and a comparison with rhodizonate tests and XRF (PDF)
Movie 1 (MP4)
Movie 2 (MP4)
Movie 3 (MP4)
