# Supplementary material for: Direct Environmental Lead Detection by Photoluminescent Perovskite Formation with Nanogram Sensitivity
Source: Environ Sci Technol. 2023 Nov 27;57(49):20494–500. doi: 10.1021/acs.est.3c06058 (PMC10720378; doi:10.1021/acs.est.3c06058)
Supplement: Supplementary file 1 — es3c06058_si_001.pdf [file es3c06058_si_001.pdf]

Supporting Information

Direct environmental lead detection by photoluminescent perovskite formation with nanogram sensitivity

Lukas Helmbrecht[1,2], Sjoerd W. van Dongen[1], Arno van der Weijden[1], Christiaan T. van Campenhout[1], Willem L. Noorduin[1,2,3*]

[1] AMOLF, Science Park 104, 1098 XG Amsterdam, The Netherlands
[2] Lumetallix B.V., Science Park 104, 1098 XG Amsterdam, The Netherlands
[3] Van 't Hoff Institute for Molecular Sciences, University of Amsterdam, Science Park 904, Amsterdam 1090 GD, The Netherlands
*E-mail: noorduin@amolf.nl

Content:

1. Photoluminescence measurement setup 1
2. List of tested materials and testing methods 2
3. Solvent screening 5
4. Different types of halide and halide mixtures 6
5. Comparison with commercial colorimetric tests 6
6. XRF testing 7
7. Analysis in luminescence variation 7
8. Description Movies 8

1. Photoluminescence Measurement Setup

The photoluminescence measurement setup utilized in this study includes the following components:

1. Excitation Source: A 10W UV LED equipped with a band-pass filter, sourced from a commercial UV flashlight, was used as the excitation source. For steady illumination, we power the LED with a constant voltage source at 4.2V. The choice to use a flashlight head instead of a specialized high-end LED for optical setups was made to mimic practical applications in field settings.

2. Imaging System: A Canon 800D camera, fitted with a Laowa 25mm f/2.8 objective, was employed for capturing images. A 420nm long-pass filter was placed in front of the objective to ensure accurate measurements. Image acquisition was conducted remotely using the EOS utility software on a computer.

3. Sample: A microscope glass slide with a 3mm diameter circle of diatomaceous earth on carbon tape was placed on a Z-Stage to allow for precise height adjustments during the experiment.

4. Environmental Control: The entire setup was housed within an aluminum box that could be closed to eliminate interference from ambient light during measurements.

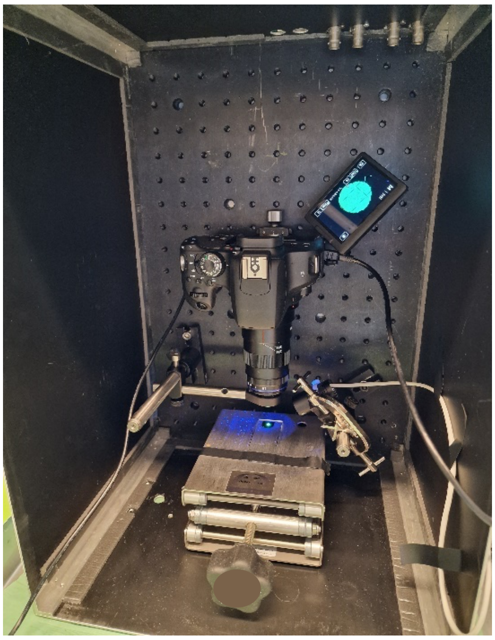

Figure S1. Photoluminescence Measurement Setup. The picture shows the light source (right), the sample on the Z-stage. The long-pass filter (mounted left) in front of the objective, the camera, and the control on the camera monitor. Not in the picture are the constant voltage source and the computer recording the image.

2. List of tested materials and testing methods

The reagent was applied using various methods (e.g. stamping, spraying, airbrushing, cotton swab). Reapplication of reagent can be used to revive the PL after decay (see for instance lead carbonate powder). Typically, PL shows instantly, but sometimes PL is after a few seconds or delayed for approximately one minute. For these screenings, luminescence was determined qualitatively by eye, and qualified as very bright, bright, less bright, dim, very dim, and speckles.

Lead-containing compounds:

Sample	Application	Luminescence	Time
Lead carbonate powder	spraying (reapplied)	very bright	instant
Lead carbonate particle film on glass	dipping	very bright	after few seconds
Lead carbonate particle film on glass	airbrush	very bright	instant
Lead carbonate particle film on glass	stamp	very bright	instant
Lead chloride	spraying (reapplied)	bright	instant
Lead white (50 mg) (hydrocerussuite, $2PbCO_3 \cdot Pb(OH)_2$, basic lead carbonate)	drip 50 µl	very bright also under flashlight	instant
Lead acetate	spraying	bright	instant
Lead nitrate (160 mg)	spraying	very dim	instant
Lead oxide	spraying (4x), reapplied through drip 100 µl	dim	after one minute
Lead white paint (fresh)	spray	very bright	instant
Lead white paint (cured)	cotton swab, soaked in reagent	very bright	instant
Metallic lead	cotton swab, soaked in reagent, reapplied as spray	less bright	delayed

	alternatively applied with a melamine sponge		
Lead glazing	spray	bright	instant
Glass 24%PbO	cotton swab, soaked in reagent, reapplied with sandpaper	less bright	increasing over the course of an hour
Rubber coating of wires	spray	very bright	instant
Lead battery cut in half	spray	bright	instant
Debris from sawing battery	spray	bright	instant
Lead-Tin (60-40) solder	spray or brush, alternatively with melamine sponge	less bright	delayed, instant with sponge
Lead fluoride	dripping	bright	delayed
Lead bromide	dripping	very bright	instant
Lead iodide	dripping	less bright, red shift	delayed
Lead(II,IV) oxide (powder)	dripping	Bright	instant
Lead primer (with Lead(II,IV) oxide)	Spray	Bright	Instant, starts blueish
NIST children's toy lead reference, 400ppm	Drip	Dim	delayed
Aluminum with 500ppm lead	Spray, reapplying	Speckles	delayed
Lead chromate (powder)	Dripping	Dim	delayed
Lead chromate paint	Dripping	Dim	instant
Lead-piping	Spray	Bright	delayed

Lead-free compounds:

Substrate	application	result	comment
Copper	Spray, cotton swab, soaked in reagent solution	no luminescence	copper is often used for waterpipes and in electronics. The test does not create false positives for copper.
Aluminium	cotton swab, soaked in reagent solution	no luminescence	no luminescence observable.
Tin	cotton swab, soaked in reagent solution	no luminescence	no luminescence observable.
Tin solder	cotton swab, soaked in reagent solution	no luminescence	tin solder is often used in electronic applications. The test does not create a false positive for tin.
SnCl2	spray	no luminescence	no luminescence observable.

TiO_2	spray	no luminescence	TiO_2 is a commonly used white pigment to replace lead. The test does not create false positives for TiO_2
ZnO_2	spray	no luminescence	ZnO_2 is a commonly used white pigment to replace lead. The test does not create false positives for ZnO_2
Glass	cotton swab, soaked in reagent	no luminescence	no luminescence observable on conventional glass for beverages.
Titanium	dripping	no luminescence	
Iron	dripping	no luminescence	
Brass	dripping	no luminescence	
Bronze	dripping	no luminescence	
Stainless Steel	dripping	no luminescence	
$CaCO_3$	dripping	no luminescence	$CaCO_3$ shows intrinsic blue luminescence prior to addition of MABr
$Ba(NO_3)_2$	dripping	no luminescence	
$Ba(OAc)_2$	dripping	no luminescence	
$BaCl_2$	dripping	no luminescence	
$BaSO_4$	dripping	no luminescence	$BaSO_4$ shows some intrinsic luminescence prior to addition of MABr
$CdSO_4$	dripping	no luminescence	
CeO_2	dripping	no luminescence	
$Cu(acac)_2$	dripping	no luminescence	
$Cu(NO_3)_2$	dripping	no luminescence	
$CuBr_2$	dripping	no luminescence	
CuCl	dripping	no luminescence	
$CuCl_2$	dripping	no luminescence	
$FeCl_2$	dripping	no luminescence	
GeI_2	dripping	no luminescence	
KBr	dripping	no luminescence	
$MgSO_4$	dripping	no luminescence	
SeO_2	dripping	no luminescence	
$SnCl_4$	dripping	no luminescence	
SnF_2	dripping	no luminescence	
SnO	dripping	no luminescence	
SnO_2	dripping	no luminescence	
Zn	dripping	no luminescence	
$Zn(OAc)_2$	dripping	no luminescence	

ZnO	dripping	no luminescence	
ZrSO4	dripping	no luminescence	
Wood	Spray	No green luminescence. Some intrinsic blue luminescence of the wood	
Poly carbonate	Spray	no luminescence	
PVC	Spray	no luminescence	
Poly styrene	Spray	no luminescence	
NIST children's toy lead reference 0 ppm	Spray	no luminescence	
Plasterboard with cardboard backing	Spray	No green luminescence. Some intrinsic blue luminescence of the cardboard	

3. Solvent screening

Solutions of MABr in different solvents were prepared. The solutions were applied onto a film of lead carbonate nanocrystals. The results are provided in the following table:

**solvent**	**Application**	**Comment**
16mg/ml MABr in IPA	3 µl with pipette	instant (less than 1s) green luminescence
10mg/ml MABr in Methanol	3 µl with pipette	green luminescence emerges during drying of the sample
10mg/mL MABr in H2O	1 µl with pipette	green luminescence emerges during drying of the sample
10mg/ml MABr in Ethanol	2 µl with pipette	green luminescence emerges during drying of the sample
5mg/ml MABr in Acetone	5 µl with pipette	green luminescence emerges during drying of the sample

18mg/ml MABr in Butanol (not fully dissolved, used supernatant)	50 µl with pipette	instant green luminescence. Less bright than with IPA.

4. Different types of halide and halide mixtures

To investigate the effect of different types of halide reagents, solutions in IPA were prepared of the following reagents: formamidium bromide (FABr), formamidium chloride (FACl), formamidium iodide (FAI), methylammonium bromide (MABr), methylammonium chloride (MACl), and formamidium iodide (FAI). The total halide concentration is typically approximately 0.16 mol/L. In the case of MACl some crystals may remain undissolved; here the supernatant is used.
The solutions were then mixed in various ratios and applied onto a lead carbonate particle film. The sample was then illuminated with a UV LED (365 nm). The observations for some specific examples are given in the following table:

Compound	Approximate molar ratio	Observed emission color
FACl-Br	Cl to Br is 6:4	blue
FABr	-	green
FABr-I	Br to I is 2:8	red
MABr-Cl	Cl to Br is 6:4	blue
MABr	-	green
MABr-I	Br to I is 2:8	red

Two further reagents were tested, as follows:

Compound	Comment	Observed emission color
Butylammonium iodide	saturate solution in IPA tested on PbCO3	green
Phenethylammonium iodide	Saturate solution in IPA tested on PbCO3	green

5. Comparison with commercial colorimetric tests

We compared the efficiency of our testing method with two commercially available tests: 3M™ LeadCheck™ Swabs and Libelyef™ Lead Test Swabs (Figure S2). Each lead test was evaluated against an ø3.2 mm circle (8 mm^2) of diatomaceous earth (sourced from https://www.naturix24.de/) loaded with lead acetate at concentrations ranging from 0.01 µg/mm^2 to 100 µg/mm^2. Lead acetate was chosen for its high detection limit in rhodizonate tests. The results were captured using a Canon 800D camera, equipped with a Sigma 17-70mm f/2.8-4 DC Macro lens, with consistent settings of ISO-200, F-Stop f/11, and 1/10 second exposure time maintained throughout the experiment.

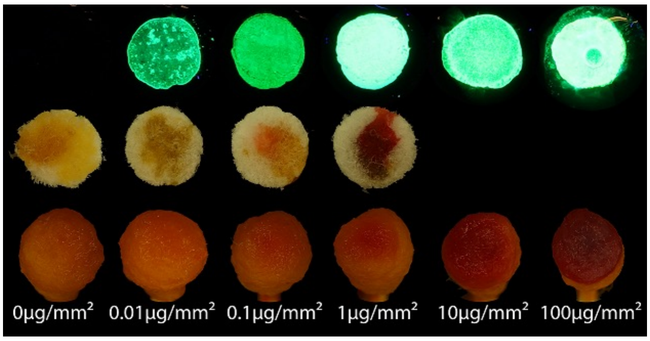

Figure S2. Comparison to colorimetric lead detection tests. For our testing method (top row), green emission indicates a positive result while the absence of light indicates a negative result. For 3M LeadCheck (middle row), a red color indicates a positive result, while yellow indicates a negative result. The Libelyef Lead Test (bottom row) also uses red for positive and yellow for negative results. Visually, 3M LeadCheck tested definitively positive at 1 $\mu g/mm^2$ and Libelyef Lead Test tested definitively positive at 10 $\mu g/mm^2$. Due to difficulty sourcing the 3M LeadCheck tests we were only able to test up to 1 $\mu g/mm^2$. We expect higher concentrations than 1 $\mu g/mm^2$ to also show a positive result for the 3M LeadCheck tests. All pictures were shot with the same camera settings and without filters for consistency and comparability.

6. XRF testing

Where feasible, the samples for Figure 4 have been tested for lead with a Niton XL5 from Thermo Fischer Scientific X-Ray Fluorescence gun set in "Mining" mode. The measurements should not be viewed as absolute results but as an indication of the amount of lead present. Diatomaceous earth samples with lead acetate were prepared akin to the experiments in Figure 2, and analyzed with XRF in "Soil" mode (Figure S3).

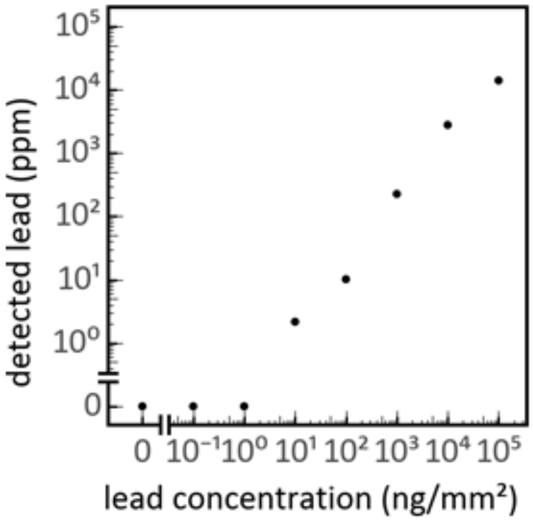

Figure S3. XRF testing. XRF measurements of diatomaceous earth samples with lead acetate, prepared akin to the experiments in Figure 2.

7. Analysis in luminescence variation

To evaluate the repeatability of our lead detection test, we conducted the PL measurement using a modified setup. We replaced the original excitation source with an array of fifteen 2W UV LEDs, placed 25 cm away from the sample to ensure uniform illumination. Imaging was carried out 30 cm from the sample using a Canon 800D camera equipped with a Sigma 17-70mm f/2.8-4.5 DC Macro lens set at a 1/20 second exposure and F5.0. No filters were applied. Samples consisted of microscope slides with carbon tape upon which a ø3.2 mm circle of diatomaceous earth was applied. These samples were loaded with 8, 80, 800, 8000 and 80000 ng of lead in the form of lead acetate in methanol. For simulating real-life large area searching, the reagent was dispensed using an atomizer spray (EAN: 5430003005477) by spraying above the samples. The resulting PL was analyzed and plotted in Figure S4. While the 1 ng/mm^2 concentration was discernible to the naked eye, the camera settings used could not capture this, indicating that the brightness drops by at least two orders of magnitude.

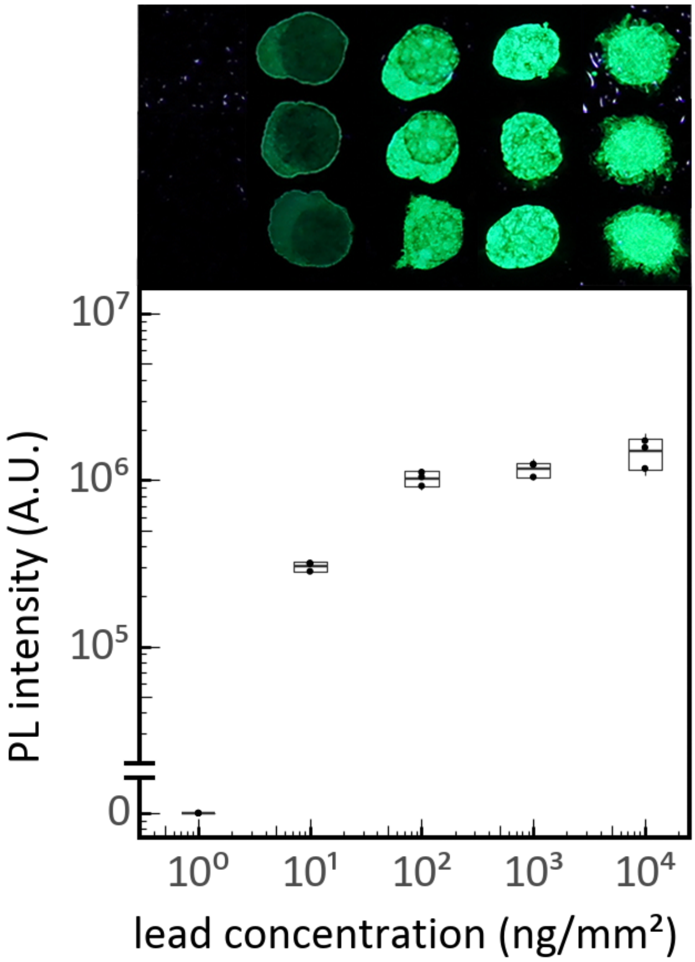

Figure S4. Analysis of PL variation. A sub-optimal PL measurement was conducted (see section 6 for setup) ranging from 1 ng/mm^2 (equivalent to 8 ng in total) to 10,000 ng/mm^2 (totaling 80,000 ng), with an exposure duration of 1/20th second and an F number of 5.0. The boxplot illustrates the data spread, with the whisker lines denoting the 95% confidence interval.

8. Description Movies

Movie 1: Real-time movie of lead detection in wineglass, showing green photoluminescence under UV-light after spraying with reagent.

Movie 2: Real-time movie of lead detection in pottery glazing, showing green photoluminescence under UV-light after spraying with reagent.

Movie 3: Real-time movie of lead detection in plastic of an electrical cable, showing green photoluminescence under UV-light after spraying with reagent.
